# Segmental Adrenal Venous Sampling in Unilateral Primary Aldosteronism With Apparent Bilateral Aldosterone Suppression

**DOI:** 10.1210/jcemcr/luae164

**Published:** 2024-09-16

**Authors:** Shotaro Miyamoto, Yuichi Yoshida, Shuhei Miyamoto, Haruto Nishida, Yoshiki Asayama, Hirotaka Shibata

**Affiliations:** Department of Endocrinology, Metabolism, Rheumatology and Nephrology, Faculty of Medicine, Oita University, Yufu City, Oita 879-5593, Japan; Department of Endocrinology, Metabolism, Rheumatology and Nephrology, Faculty of Medicine, Oita University, Yufu City, Oita 879-5593, Japan; Department of Radiology, Faculty of Medicine, Oita University, Yufu City, Oita 879-5593, Japan; Department of Diagnostic Pathology, Faculty of Medicine, Oita University, Yufu City, Oita 879-5593, Japan; Department of Radiology, Faculty of Medicine, Oita University, Yufu City, Oita 879-5593, Japan; Department of Endocrinology, Metabolism, Rheumatology and Nephrology, Faculty of Medicine, Oita University, Yufu City, Oita 879-5593, Japan

**Keywords:** primary aldosteronism, unilateral primary aldosteronism, adrenal venous sampling, AVS, segmental adrenal venous sampling, S-AVS

## Abstract

Apparent bilateral adrenal suppression (ABAS), where aldosterone/cortisol ratios in both adrenal veins are lower than in the inferior vena cava, yields uninterpretable adrenal venous sampling (AVS) results and is poorly understood. A 57-year-old male with hypertension and spontaneous hypokalemia was admitted to our hospital. Confirmatory tests established a diagnosis of primary aldosteronism (PA). Initial AVS indicated ABAS, but unilateral PA remained possible due to elevated aldosterone, low renin, hypokalemia, and a right adrenal nodule (8 × 7 mm) on computed tomography. Subsequently, a second, super-selective AVS identified tributaries from areas of aldosterone hypersecretion, enabling accurate localization of unilateral PA. ABAS may occur due to anatomical factors such as dilution by tributaries from nonaldosterone-producing adenoma (APA) areas with suppressed aldosterone production. Super-selective AVS proves beneficial in diagnosing unilateral PA concealed within ABAS by pinpointing tributaries from APA regions.

## Introduction

Adrenal venous sampling (AVS) is the gold standard for localizing primary aldosteronism (PA), determined by comparing the aldosterone/cortisol (A/C) ratio from the central vein of the affected adrenal gland with that of the contralateral adrenal gland and the inferior vena cava [1]. Despite its critical role, AVS is technically demanding, requiring considerable expertise from the radiologist, and, even when successfully performed, some results remain difficult to interpret. Apparent bilateral adrenal suppression (ABAS), with lower A/C ratios in both central adrenal veins than in the inferior vena cava [2], can be addressed by segmental adrenal vein sampling (S-AVS), which samples from all accessible branches of the adrenal veins for a more detailed analysis of intra-adrenal aldosterone secretion [3], particularly in distinguishing between bilateral aldosterone-producing adenoma (APA) and idiopathic hyperaldosteronism [4]. This report describes a case of right unilateral PA initially presenting as ABAS in conventional AVS but subsequently confirmed as right unilateral PA through S-AVS, highlighting the clinical value of S-AVS when central AVS (C-AVS) does not conclusively localize the condition.

## Case Presentation

A 45-year-old man, diagnosed with hypertension, began antihypertensive medication at age 50. Seven years later, his blood pressure was 135/84 mmHg, and a blood test indicated spontaneous hypokalemia [potassium (K), 2.4 mmol/L; normal reference range (RR), 3.6-4.9 mmol/L], prompting the initiation of oral potassium supplementation (7.2 mEq/day), which was gradually increased to a final dose of 32.4 mEq/day. He was subsequently admitted to our hospital for further evaluation of PA.

## Diagnostic Assessment

**Figure 1.**
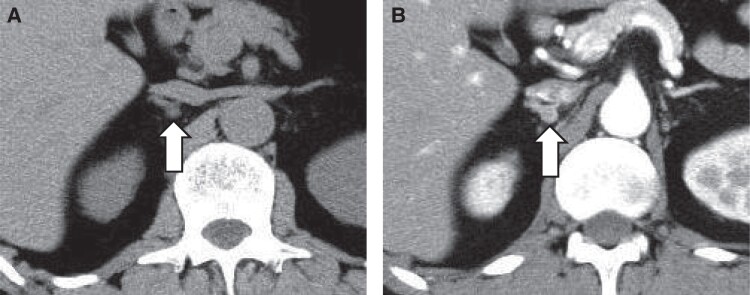
(A) Plain CT image and (B) iodine contrast CT image showing a right adrenal nodule (8 × 7 mm) with CT attenuation values of < 10 HU. Abbreviation: CT, computed tomography.

**Figure 2.**
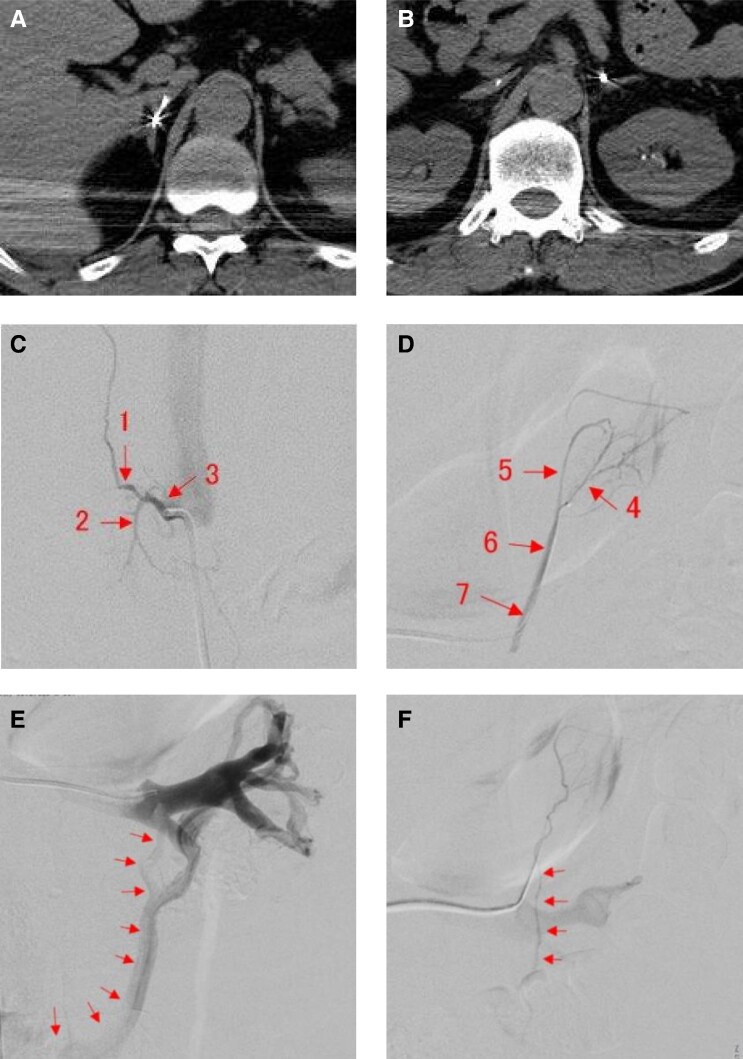
CT angiographic images from the second S-AVS. (A) Guidewire insertion into the right adrenal vein. (B) Guidewire insertion into the left adrenal vein. (C) 1: upper tributary; 2: lower tributary; 3: central vein of the right adrenal vein. (D) 4: superolateral tributary; 5: superomedial tributary; 6: common trunk; 7: central vein of the right adrenal vein. (E) Contrast angiography of the left renal vein showing the circumaortic vein draining into the inferior vena cava. (F) An inferior emissary vein seen in contrast from branch 4 was suspected to flow into this vessel. Abbreviations: CT, computed tomography; S-AVS, segmental adrenal vein sampling.

PA was suspected due to a high aldosterone-to-renin ratio (96.6 ng/dL per ng/mL/h) (plasma renin activity .3 ng/mL/h; RR, .2-2.7 ng/mL/h) and plasma aldosterone concentration (PAC) [29.01 ng/dL (803.78 pmol/L); RR, 3.0-15.9 ng/dL (83.2-441.1 pmol/L)]. The diagnosis of PA was confirmed through a positive captopril challenge test (active renin concentration 1.2 pg/mL; RR, 3.2-36.3 pg/mL), PAC 26.47 ng/dL(734.38 pmol/L), aldosterone-to-renin ratio 22.06 (≥4), a positive saline loading test [PAC 30.43 ng/dL (844.95 pmol/L) after 2 hours (≥6 ng/dL), and a positive oral salt loading test [24-hour urinary aldosterone 16.5 μg/day (>6 μg/day) and 24-hour urinary Na 347.9 mEq/day (>170 mEq/day)] [5]. An abdominal computed tomography (CT) scan revealed a right-sided adrenal nodule (8 × 7 mm) with CT attenuation values of <10 HU (Fig. 1). Cortisol diurnal variation was normal, and a 1 mg dexamethasone suppression test indicated cortisol suppression to 23.79 nmol/L [.861 μg/dL; RR, < 49.7 nmol/L (<1.8μg/dL)], excluding autonomous cortisol secretion. The 24-hour urinary catecholamines [epinephrine, 16.3 μg/day (<41); norepinephrine, 168 μg/day (<160); dopamine, 715.5 μg/day (<1100)] and urinary fractionated metanephrines [metanephrine, .20 mg/day (<.20) and normetanephrine, .29 mg/day (<.28)] did not exceed threefold the upper limit of the normal range, ruling out a pheochromocytoma complication. AVS was conducted under ACTH stimulation (Cosyntropin 250μg, intravenous bolus), and the results were interpreted according to established guidelines [5]. Successful catheter insertion was verified by comparing cortisol levels in the adrenal vein with those in the inferior vena cava. To determine lateralization, the A/C ratio was calculated; bilateral PA was diagnosed when the lateralization ratio (LR) was 1.09 < 4, but the A/C ratios of both left and right central adrenal veins were lower than those in the inferior vena cava (contralateral ratio <1). Due to the presence of spontaneous hypokalemia and a right adrenal nodule, the possibility of unilateral PA persisted, prompting a second, super-selectively performed AVS under ACTH stimulation (Fig. 2). This AVS showed that the A/C ratios in the bilateral central adrenal veins were suppressed, like the initial C-AVS, but the A/C ratio in the right lower tributary was elevated at 30.9. Applying the A/C ratio from the right upper adrenal vein and the left central adrenal vein, the LR was 5.96 (>4 is consistent with unilateral disease), and the contralateral ratio was .18 (<1 is consistent with unilateral disease) [5], leading to the diagnosis of right unilateral PA (Table 1).

**Table 1.** The first C-AVS and the second S-AVS under ACTH stimulation

	First C-AVS			Second S-AVS		
	Aldosterone	Cortisol	A/C ratio	Aldosterone	Cortisol	A/C ratio
Right						
Upper tributary				178.17 ng/dL (4943 pmol/L)	357.0 µg/dL (9856 nmol/L)	.50
Central vein	95.08 ng/dL (2638 pmol/L)	202.0 µg/dL (5577 nmol/L)	.47	478.14 ng/dL (13 264 pmol/L)	424.0 µg/dL (11 710 nmol/L)	1.13
Lower tributary				1324.72 ng/dL (36 744 pmol/L)	428.0 µg/dL (11 801 nmol/L)	3.1
Left						
Upper internal				139.77 ng/dL (3874 pmol/L)	382 µg/dL (10 539 nmol/L)	.37
Upper lateral				256.74 ng/dL (7125 pmol/L)	517 µg/dL (14 263 nmol/L)	.50
Central vein	125.50 ng/dL (3480 pmol/L)	243.0 µg/dL (6698 nmol/L)	.52	158.82 ng/dL (4405 pmol/L)	306.0 µg/dL (8419 nmol/L)	.52
Common trunk				200.50 ng/dL (5562 pmol/L)	277.0 µg/dL (7630 nmol/L)	.72
Peripheral						
Inferior vena cava (L4)	59.19 ng/dL (1643 pmol/L)	18.7 µg/dL (515 nmol/L)	3.17	61.83 ng/dL (1715 pmol/L)	22.4 µg/dL (618 nmol/L)	2.76
Inferior vena cava (L2)				59.30 ng/dL (1645 pg/mL)	22.5 µg/dL (620 nmol/L)	2.63
Inferior vena cava (L3)				67.03 ng/dL (1858 pmol/L)	24.6 µg/dL (678 nmol/L)	2.73
Left femoral vein				56.08 ng/dL (1556 pmol/L)	22.5 µg/dL (621 nmol/L)	2.49
Localization diagnosis		Bilateral			Right	

Abbreviations: A/C, aldosterone/cortisol; C-AVS, central adrenal vein sampling; L, lumbar; S-AVS, segmental adrenal vein sampling.

## Treatment

Following the examination, the patient commenced treatment with spironolactone 25 mg and continued oral K supplementation for hypokalemia until surgery. He then underwent a laparoscopic right adrenalectomy. The postoperative course was uneventful, and both spironolactone and oral K supplementation were discontinued.

## Outcome and Follow-up

**Figure 3.**
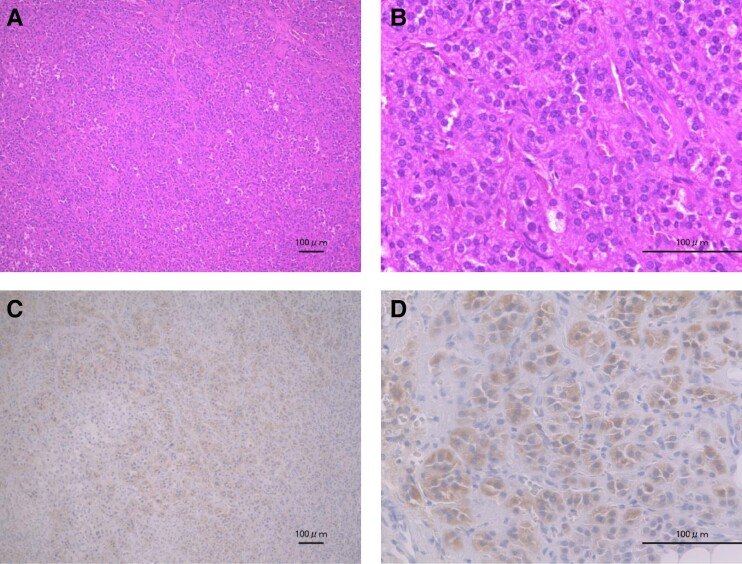
Microscopic findings of an aldosterone-producing adenoma. (A) and (B) HE staining shows tumor cells arranged in nests with a slightly elevated nucleus-to-cytoplasm ratio, rounded, chromatin-rich nuclei, and mixed cells with pale cytoplasm: (A) at 100× magnification; (B) at 400× magnification. (C) and (D) Immunohistochemical staining positive for CYP11B2: (C) at 100× magnification; (D) at 400× magnification.. The English in this document has been checked by at least 2 professional editors, both native speakers of English. For a certificate, please see http://www.textcheck.com/certificate/PFvI5h. Abbreviation: HE, hematoxylin-eosin.

A small nodular tumor within the resected right adrenal gland was noted. The tumor cells exhibited no malignant features, such as division or necrosis, which consist of CYP11B2-positive cells, confirming the diagnosis of aldosterone-producing adrenal cortical adenoma (Fig. 3). In addition, the CYP11B2-positive cells were also found in the zona glomerulosa of the adjacent adrenal tissue (aldosterone-producing micronodules). One-month postsurgery, the patient's blood pressure was 126/84 mmHg without antihypertensive medications, with K 4.27 mmol/L, PAC 7.17 ng/dL (198.95 pmol/L), and active renin concentration 5.0 pg/mL, all showing marked improvement. One year after surgery, his blood pressure and K levels remained well controlled, with no recurrence of PA.

## Discussion

This report demonstrates that ABAS can occur in unilateral PA due to the dilution effect from tributaries originating in non-APA areas when using the central vein A/C ratio and highlights the use of S-AVS in localizing such cases.

In our case, both the initial and repeat AVS indicated ABAS, where the A/C ratios of the left and right central adrenal veins were suppressed compared to the inferior vena cava. Wolley et al reported ABAS in 2.6% of patients undergoing AVS, with 10 of 22 patients diagnosed with unilateral PA after repeated AVS under ACTH stimulation, suggesting ABAS could relate to sampling during a quiescent phase of aldosterone secretion from APA, potentially reduced by repeated AVS under ACTH stimulation [6]. Another potential cause for ABAS is incidental deep sampling during right central adrenal vein sampling [7]. The right central adrenal vein is narrower, has a shorter trunk than the left, and directly enters the inferior vena cava posterolaterally [8, 9], complicating sampling. Adjustments by the radiologist or thoracic movements during respiration can inadvertently push the catheter into respective tributaries. In unilateral PA, aldosterone production in the contralateral adrenal gland is suppressed, and even within the lesioned adrenal gland, there can be segments with excess aldosterone production (APA) and areas with suppressed aldosterone production (non-APA) [7]. If the catheter tip extends beyond the branch confluences into the central vein, incidental deep sampling from a non-APA tributary can occur, suppressing the A/C ratio of the central vein on both the lesioned and normal sides. Although rare, ectopic aldosterone production and abnormal adrenal vein anatomy can also contribute to ABAS [10, 11], but such cases are extremely uncommon.

We initially suspected incidental deep sampling from the non-APA segment when sampling the right central adrenal vein. Particularly during the second S-AVS, the catheter was advanced into the lower tributary and retracted after sampling at the same site to ensure that the tip was in the central adrenal vein, verified by contrast medium before sampling. Table 1 shows that the A/C ratio of the right central vein was lower than that of the lower tributary but higher than the upper tributary, suggesting that the blood sampled in the right central vein was mixed from the upper and lower branches. These findings make incidental deep sampling unlikely during the second AVS of the right adrenal central vein.

Next, we considered collateral blood vessels from the APA segment flowing into the inferior vena cava, potentially elevating the A/C ratio in that area and leading to ABAS. Figure 2E and F depict collateral blood flow from the left renal vein, connecting to the branch of the left adrenal vein and draining into the inferior vena cava, possibly contributing to the elevated A/C ratio of the inferior vena cava. However, no collateral blood flow from the right adrenal vein on the APA side into the inferior vena cava was found. Furthermore, blood samples from 3 distinct lumbar levels of the inferior vena cava and the right femoral vein all showed higher A/C ratios compared to the right and left central adrenal veins (Table 1). Even if collateral blood flow from the APA segment existed, it was unlikely to extend peripherally beyond the right femoral vein, ruling out ABAS due to collateral blood flow. The possibility of an ectopic aldosterone-producing tumor was also excluded since PA improved post-right adrenalectomy.

We then hypothesized that the dilution effect of tributaries from the non-APA segment lowered the A/C ratio in the right central vein, resulting in ABAS. S-AVS offers more accurate localization of aldosterone hypersecretion by direct sampling from the tributary of the APA segment, minimizing the dilution effect from the non-APA segment. In our case, compared to the A/C ratio of the right central adrenal vein, the LR was <4, not meeting the criteria for localization. However, by applying the A/C ratio in the lower tributary closer to the APA and reducing the dilution effect, the LR far exceeded the criteria (Table 1). This case highlights the use of S-AVS in diagnosing unilateral PA with ABAS. However, although S-AVS provides more accurate localization than C-AVS, it does not necessarily pinpoint the exact APA site, and thus we do not recommend S-AVS results to support partial adrenalectomy for APA.

AVS is recognized as the gold standard for the localization diagnosis of PA, but due to its invasiveness and technical difficulty, scoring methods have been proposed to predict the subtype of PA without performing AVS [12]. Although these methods have not yet been widely adopted in clinical practice, many endocrinologists typically use the results of blood K, PAC, and confirmatory tests to predict the subtype of PA before undertaking AVS. Song et al reported that in cases of florid PA with markedly elevated aldosterone, suppressed renin, hypokalemia, and a clear unilateral adenoma on CT, AVS may be unnecessary [13]. Additionally, Umakoshi et al found that in patients under 35 years old with unilateral findings on CT and hypokalemia, AVS consistently revealed unilateral hyperaldosteronism [14]. In this case, we anticipated unilateral localization based on the positive results of all confirmatory tests, spontaneous hypokalemia, and the right adrenal nodule evident on CT, but since the patient was well over 35 years of age, we decided to perform AVS. Contrary to expectations, the result indicated bilateral involvement. Subsequently, we shared the clinical findings of PA and our localization expectations with the radiologist, suspecting unilateral PA undetected by C-AVS, leading us to perform S-AVS. It is important to note that, unlike previous reports [6, 15], repeated C-AVS alone did not result in a localization diagnosis. Given its cost and complexity, performing all AVS super-selectively is impractical. However, when the endocrinologist's prediction of PA localization does not align with the C-AVS results or there is a strong suspicion of unilateral PA before AVS, S-AVS should be considered to avoid a missed diagnosis of unilateral PA. Effective collaboration and information sharing with the radiologist are essential in making this decision.

In conclusion, we encountered a case of unilateral PA with ABAS where S-AVS proved valuable in achieving accurate localization. Clinicians should consider S-AVS when conventional AVS results in ambiguous findings to prevent overlooking unilateral PA.

## Learning Points

- S-AVS is effective for localizing unilateral PA when ABAS is observed on C-AVS.
- ABAS can occur in unilateral PA due to the dilution effect of tributaries from non-APA segments, even in the absence of incidental deep sampling or anatomical abnormalities such as collateral blood flow.
- Collaboration between endocrinologists and radiologists is essential for determining the necessity of S-AVS.

## Data Availability

Original data generated and analyzed for this case report are included in this published article.
